# Supramolecular Assembly of Plant Cell Wall-Derived Cellulose Nanosheets with Polyacrylamide for Sustainable Sand Stabilization

**DOI:** 10.3390/polym18101188

**Published:** 2026-05-13

**Authors:** Feifan Xie, Xiaoyan Zha, Xiaoxuan Guo, Zongying Fu, Yun Lu

**Affiliations:** Research Institute of Wood Industry, Chinese Academy of Forestry, Beijing 100091, China; ff66997757@163.com (F.X.); 18406938427@163.com (X.Z.); guoxx901@163.com (X.G.);

**Keywords:** sand fixation, nanocellulose, supramolecular assembly

## Abstract

To address the global challenge of desertification, it is essential to develop sustainable and biodegradable materials for sand fixation to support ecological restoration in arid regions. In this work, a CNS/PAM biocomposite system was constructed through the supramolecular assembly of highly flexible two-dimensional cellulose nanosheets (CNS) and polyacrylamide (PAM). Benefiting from the flexible layered structure of CNS and the abundant hydroxyl and carboxyl groups on their surface, a conformal coating and an interparticle bridging network were formed via hydrogen bonding and coordination interactions with mineral cations. The introduction of PAM further regulated the hydrogen-bonding network, which improved structural uniformity and mechanical integrity. The resulting composites showed strong resistance to both wind and water erosion (erosion loss < 0.1%) and reached a compressive strength of up to 0.23 MPa, while maintaining good environmental compatibility. This study clarifies the structure–interaction–property relationships of cellulose nanosheet-based supramolecular assemblies and provides a new theoretical basis and practical pathway for designing biodegradable sand-fixing materials.

## 1. Introduction

Currently, driven by human activities, global desertification has become a serious environmental issue that threatens ecosystem stability, food security, and sustainable development [1,2,3]. This situation highlights the urgency of developing materials for sand fixation and environmental restoration to control or reduce land desertification. Compared with plant-based or mechanical methods, chemical sand stabilization is more effective and easier to apply [4]. This method works by spraying stabilizing agents onto loose sand surfaces to form a film, which improves soil properties and binds dispersed sand particles together [5]. However, the high cost, difficulty in recycling, and environmental impact of current inorganic and organic sand-stabilization materials limit their widespread use. Consequently, there is a pressing need for an economical, effective, and nature-based solution [6,7,8,9]. Developing sand-stabilization materials that are efficient, easy to use, and environmentally friendly is of great importance.

Cellulose, the most abundant natural polymer on Earth, possesses a unique hierarchical fiber structure and strong fiber–interface interactions. These features give biomaterials excellent structural properties and have attracted wide research interest [10,11,12,13]. In particular, nanocellulose has gained significant attention due to its wide availability, excellent mechanical strength, and eco-friendliness, making it an ideal matrix for films [14,15,16]. Severely desertified regions contain large amounts of weakly cohesive sandy soil, which is easily blown away by wind. When the lift and drag forces exceed the combined effects of gravity, cohesion, and friction on each particle, sand and gravel begin to move [17,18,19]. Flexible cellulose and hemicellulose fragments can bend and entangle to form dense network structures, which help aggregate soil and gravel particles [20,21,22]. Traditional one-dimensional nanocellulose materials (CNF) mainly rely on forming a network structure to fix sand and gravel particles through a bridging effect. However, due to their limited mechanical strength and high hygroscopicity, these structures are often unstable [23,24]. Based on the abundant surface chemical structures of nanocellulose, further functionalization can be achieved through modification or by combining with polymers like polyurethane (PU) and polyvinyl alcohol (PVA). These features make CNF-derived sand-fixing agents promising for ecological sand fixation [25,26].

For example, carboxymethyl cellulose (CMC) is often used as a single-component sand-fixing material. It can form a coating film on the surface of sand particles, which reduces water infiltration and evaporation. However, its application is limited by poor mechanical performance, high hygroscopicity, and a limited ability to improve the mechanical properties of sandy soil [27]. To further improve the performance of cellulose-based sand-fixing materials, polymer matrices have become ideal candidates for composites. Among them, polyacrylamide (PAM) shows great potential for sand-fixing. PAM contains abundant amide groups and has high viscosity and hydration properties. It can adsorb, wrap, and bond soil particles, improving the structure of soil particles, increasing soil aggregation, and reducing water and wind erosion [28,29].

In this study, natural nanosheet materials were extracted from plant cell walls. Due to their unique morphology, cellulose nanosheets (CNS) can achieve a higher packing density and exhibit superior mechanical properties than CNF [30]. Unlike traditional nanocellulose sand-fixing materials that rely on linear point contacts, CNS forms a more effective interlocking structure through a wrapping mechanism and surface-to-surface contact. With the inherent adhesion of cell wall components and their electrostatic affinity for metal ions, these nanosheets can form coordination and chelation structures with mineral sand and gravel through metal-ion-mediated interactions. At the microscale, the high flexibility of CNS allows close contact with the surface of sand and gravel, enabling adaptive interfacial interactions and stable particle fixation [31]. The introduction of polyacrylamide (PAM) further reorganizes the hydrogen-bonding network, changes the interfacial interactions within the composite, and improves its strength through electrostatic and hydrogen-bonding interactions. By exploring the structure–property–function relationship of this system, this work aims to provide new insights for sustainable design and offer new possibilities for utilizing waste biomass as stabilization agents.

## 2. Materials and Methods

### 2.1. Materials

2,2,6,6-tetramethylpiperidine-1-oxyl (TEMPO), Sodium hypochlorite (NaClO, 6% active chlorine), sodium chlorite (NaClO_2_, 80%), polyacrylamide (PAM), potassium hydroxide (KOH), nanosilica (SiO_2_), and citric acid (C_6_H_8_O_7_) were purchased from Shanghai Macklin Biochemical Co., Ltd. (Shanghai, China). Oxidized cellulose nanofibers (TOCNF) and carboxymethyl cellulose (CMC) were obtained from Aladdin Reagent Co., Ltd. (Shanghai, China). Graphene was purchased from Suzhou Hengqiu Technology Co., Ltd. (Suzhou, China). Ethanol was supplied by Beijing Chemical Plant (Beijing, China). China fir (*Cunninghamia lanceolata*) wood blocks (20 × 20 × 20 mm) were commercially obtained. Deionized water was used in all the preparations.

### 2.2. Synthesis of Cellulose Nanosheet

First, the wood was crushed and sieved through a 20-mesh screen. A total of 2 g wood powder was mixed with 2 wt% KOH solution and stirred at 90 °C for 3.5 h. After filtration, the solid was added to 2 wt% NaClO and stirred at 80 °C for 2 h. Then, NaClO, TEMPO, and NaClO_2_ were added separately, and the mixture was reacted at 80 °C for 2.5 h. The reaction was quenched using ethanol. Finally, the product was thoroughly washed with deionized water, dispersed in deionized water, and sonicated for 40 min under ice-bath conditions [32]. Well-dispersed CNS were obtained.

### 2.3. Preparation of PN Sand Dispersion

First, CNS solutions with different concentrations (0.1%, 0.2%, and 0.4%) were prepared, followed by the addition of 0.2% PAM. The mixture was stirred at room temperature for 12 h to ensure complete dissolution and degassing, yielding the composite sand-fixing agent, denoted as PN. Based on the CNS content, the samples were labeled as PN1 (0.1% CNS), PN2 (0.2% CNS), and PN4 (0.4% CNS).

### 2.4. Microstructure and Chemical Properties Analysis

The microstructure of the cellulose nanosheets and the consolidated layer was characterized using field emission scanning electron microscopy (JEOL, Tokyo, Japan) and transmission electron microscopy (FEI, Hillsboro, OR, USA). The chemical composition of the sand-fixing agents was analyzed by Fourier transform infrared spectroscopy (Thermo Fisher Scientific, Waltham, MA, USA) and Raman spectroscopy (HORIBA Scientific, Kyoto, Japan). The crystal structure was examined using X-ray diffraction (Rigaku, Akishima, Japan). Atomic force microscopy (Asylum Research, Santa Barbara, CA, USA) was employed to characterize the thickness and nanomechanics of the nanosheets. The changes in electron cloud density of the sand-fixing agents were investigated using X-ray photoelectron spectroscopy (Thermo Fisher Scientific, East Grinstead, UK). The morphology was observed using an optical microscope (Olympus, Tokyo, Japan) and a super-depth microscope (KEYENCE, Osaka, Japan). The hydrophilicity of the consolidated layer was evaluated using an OCA20 contact angle/surface tension analyzer (Dataphysics, Stuttgart, Germany). The tensile strength of the consolidated layer films was measured using a KXWW-01C mechanical testing machine (Kebiao Testing Instrument Manufacturing Co., Ltd, Chengde, China). The rheological properties of the samples were measured using a HAAKE MARS60 rheometer (HAAKE, Karlsruhe, Germany).

### 2.5. Sand Fixation Test

First, approximately 100 g of sand samples was filled into a disk-shaped container and piled into a cone shape. Solutions with different concentrations were sprayed uniformly onto the surface of the sand specimens at a rate of 3 L/m^2^. The treated specimens were then placed in an oven at 40 °C for 72 h. After a surface crust formed, the experimental sand cones were obtained.

For the wind erosion test, the wind speed was measured using an anemometer. The test was conducted at a wind speed of 12 m/s for 5 min, and the mass loss of the final sand-fixing system was recorded.

The water erosion test was performed by simulating rainfall using water (10 L/m^2^). The experiment lasted for 15 min, and the weight loss was recorded every 5 min. The eroded sand samples were collected, dried, and weighed.

In total, 25 g of sand was mixed evenly with 10 mL of sand fixing solution, and then the mixture was cast into a mold (height 2.2 cm, diameter 2.0 cm). The sample was dried in an oven at 40 °C for 3 days, and then the KXWW-01C mechanical testing machine was used to perform compressive testing on the obtained sand core. All experiments were performed with a minimum of three independent replicates.

### 2.6. Growth Retardation Experiment

Sow wheat (*Triticum aestivum* L.) in sandy soil, then spray 3 L/m^2^ of different sand-fixing agent solutions on the surface of the soil, and monitor the development of the plants.

## 3. Results and Discussion

### 3.1. Bio-Inspired Design and Sand-Stabilization Behavior

**Figure 1:**
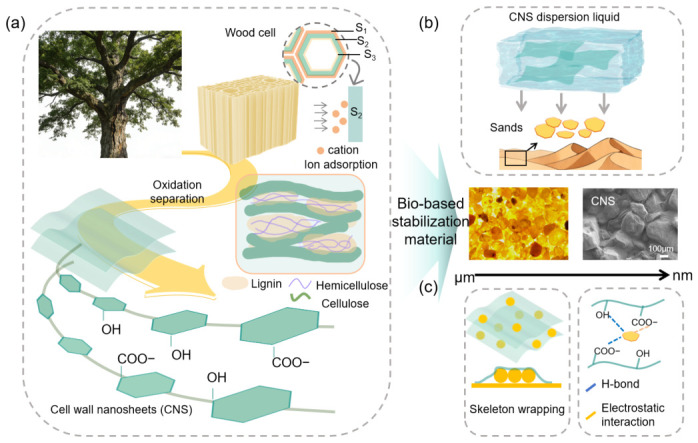
Schematic illustration of the bio-based sand-stabilization strategy and its applications: (**a**) exfoliation of cell wall-derived nanosheets; (**b**) schematic of sand-stabilization application and surface consolidation of sand and gravel particles before and after treatment; (**c**) schematic illustration of the sand-stabilization mechanism.

The complete preparation process of the sand-fixing agent is shown in Figure 1. In natural plant cell walls, a complex and efficient response to heavy metal stress is formed through the synergy of multiple components, including the adsorption, transport, and mineral deposition of metal ions [33]. Inspired by this effect, we designed a biomimetic strategy based on cell wall structures for sand fixation. In our previous work, we confirmed the presence of CNS within plant cell walls and successfully obtained them [32,34].

As shown in Figure 1a, CNS are derived from renewable lignocellulosic biomass and retain the β-1,4-glucan backbone. In addition to the naturally abundant hydroxyl groups, carboxyl groups are introduced during the oxidation and separation process. These nanosheets have a high aspect ratio and a layered hydrogen-bonding framework, which provide mechanical strength and interfacial reactivity. The practical application of these nanosheets in sand stabilization is shown in Figure 1b. At the microscale, dispersed particles are effectively wrapped and fixed. This stabilization results from the combined effects of physical confinement and chemical interactions (Figure 1c). Specifically, the two-dimensional morphology of the nanosheets enables strong adhesion and conformal wrapping of the particles, while electrostatic interactions and the inter-nanosheet hydrogen-bonding network further enhance the structural stability of the stabilized layer.

### 3.2. Physicochemical Characteristics of CNS

**Figure 2:**
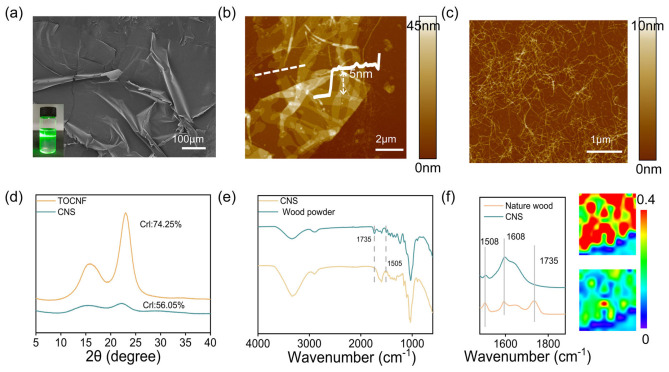
CNS structural characteristics and chemical components. (**a**) SEM images and solution images of CNS; (**b**) AFM images and thickness of CNS; (**c**) AFM image of CNF; (**d**) XRD image of CNS; (**e**) FTIR image of CNS; (**f**) two-dimensional infrared image of surface functional groups of CNS.

As shown in Figure 2a and Figure S1a, the CNS exhibit a sheet-like structure with abundant wrinkles, and the surface undulations demonstrate their good flexibility [35]. The dispersion shows a clear Tyndall effect, suggesting good stability. No aggregation or sedimentation is observed even after 60 days (Figure S1b). The excellent dispersion is mainly due to the electrostatic repulsion provided by the carboxyl groups introduced during oxidation. Acid–base titration shows that the carboxyl content is about 0.44 mmol/g, and the zeta potential of the dispersion reaches −36.6 mV (Figure S1c), collectively confirming the strong electrostatic stabilization of the nanosheets [36].

The thickness of CNS was measured by AFM. The results show a uniform thickness, with a single-layer step height of about 5 nm (Figure 2b). As shown in Figure 2c, the AFM image of traditional nanocellulose (CNF) presents a one-dimensional linear structure, which is very different from the morphology of CNS. This difference mainly comes from their structural composition. During the preparation of CNF, hemicellulose and lignin are removed more completely, and stronger mechanical treatment breaks the hydrogen bonding and other interactions between cellulose chains [37]. In contrast, CNS retains part of the hemicellulose and lignin. Component analysis shows that CNS is mainly composed of cellulose (55.4%) (Figure S1d), along with hemicellulose (14%) and lignin (11.1%) (Figure S1d). The adhesion of these interlayer components helps maintain the two-dimensional structure. As shown in Figure S2a, TEM images reveal a clear sheet-like structure of CNS with good flexibility and the ability to bend. HRTEM further shows its crystal structure, indicating a distinct two-phase feature. Amorphous components such as hemicellulose are attached to the surface of oriented cellulose. The lattice spacing is about 0.386 nm, corresponding to the (002) plane of cellulose (Figure S2b) [38].

The XRD pattern shows typical cellulose type I characteristics (Figure 2d). The crystallinity is about 56.05%, significantly lower than CNF (74.25%). This further confirms that the presence of hemicellulose and other matrix components affects the structural features.

Further structural insights are provided by Fourier transform infrared spectroscopy (FTIR) in Figure 2e. The disappearance of the peak at 1735 cm^−1^, which is assigned to the acetyl groups of hemicellulose, and the reduced intensity of the lignin C=C vibration near 1505 cm^−1^ indicate the depolymerization of hemicellulose and lignin. This process helps separate cellulose nanosheets from the plant cell wall matrix [39,40]. To further elucidate the surface chemical structure of CNS, two-dimensional infrared spectroscopy was employed for chemical imaging. The spatial overlap between the characteristic bands of cellulose and carboxylate groups suggests that oxidation preferentially occurs on cellulose-rich surface regions (Figure 2f) [41].

**Figure 3:**
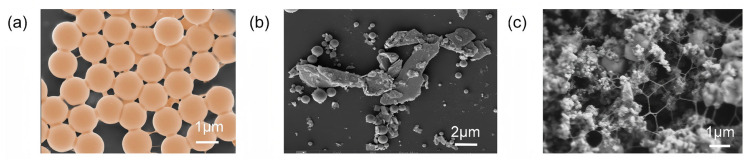
SEM images of two-dimensional flexible CNS (**a**), graphene (**b**), and nanocellulose (**c**) immobilized nanosilica in CNS.

To elucidate the structural advantages of CNS in particle fixation, their interfacial behavior was compared with typical two-dimensional and one-dimensional materials. Dispersions of CNS, graphene, and CNF were sprayed onto the surface of nanosilica, as shown in Figure 3. Compared with graphene, its high bending stiffness limits its ability to adapt to microscale surface roughness, resulting in limited wrapping of nanosilica. In contrast, CNS has lower bending stiffness, which allows close interfacial contact and stronger mechanical interlocking. As a result, CNS can more effectively wrap and fix nanosilica particles with uniform surfaces [42]. CNF mainly forms linear contact and network structures with nanosilica, and it tends to form larger aggregates due to interlocking. In comparison, the planar geometry of CNS provides a larger contact area, reduces interfacial stress concentration, and enables more uniform particle encapsulation, thereby improving load transfer efficiency.

The two-dimensional CNS architecture promotes a hierarchical hydrogen-bonded stacking network, providing strong interlayer cohesion while allowing interfacial sliding. As shown in Figure S3a,b, the fracture surface of the CNF film is relatively flat, whereas the CNS film exhibits a sheet-like, jagged topography with tightly stacked layers. The pull-out of these layers effectively buffers the fracture process, contributing to a tensile strength of 102 MPa for the CNS cured film, while the CNF film is approximately 76 MPa [43]. Figure S3c demonstrates that CNS possess excellent mechanical properties compared to other nanofiber materials and petrochemical plastics, ensuring the stability of the consolidated layer. The combination of excellent mechanical flexibility at the microscale and robust mechanical performance at the macroscale endows the CNS with significant potential for sand fixation.

### 3.3. Composite Sand-Fixing Agent Supramolecular Network

**Figure 4:**
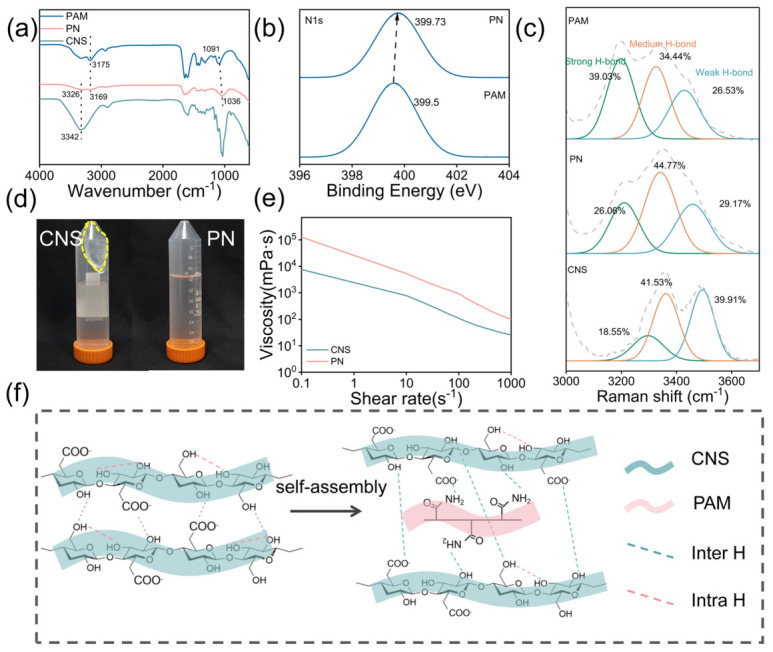
Chemical structure of composite sand-fixing agent (PN). (**a**) FTIR spectra of CNS, PAM, and mixture (PN); (**b**) N1s peak separation of PAM and PN; (**c**) Raman peaks of CNS, PAM, and PN; (**d**) the precipitation of CNS and PN before and after centrifugation; (**e**) rheological curves of CNS and PN solutions; (**f**) CNS and PAM composite diagram.

PAM and CNS (PN) can form highly ordered supramolecular networks through multiple, synergistic non-covalent hydrogen bonds. The FTIR spectrum (Figure 4a) reveals a strong characteristic peak at 1650 cm^−1^, corresponding to the stretching vibration of C=O in the side chains of PAM, and a peak at 1352 cm^−1^ corresponding to the bending vibration of N–H. These results confirm that PAM chains are successfully introduced into the composite system. For CNS, the broad peak at 3342 cm^−1^ is attributed to the -OH stretching vibration from the cellulose backbone and surface carboxyl groups, while pure PAM shows a characteristic peak at 3175 cm^−1^ from the asymmetric stretching of -NH_2_. After the composite formation, both of these characteristic peaks exhibit a significant redshift, shifting to 3326 cm^−1^ and 3169 cm^−1^. This shift indicates strong intermolecular hydrogen bonding between the hydroxyl and carboxyl groups on the CNS surface and the amide groups on the PAM chains [44,45,46]. These results show that CNS is not merely physically mixed but is anchored within the PAM network through multiple hydrogen bonds, leading to a reconstructed network from one-dimensional chain entanglement to a two-dimensional sheet-reinforced structure. As shown in Figure 4b, in the N1s spectra of the composite, the binding energy increases from 399.5 eV to 399.73 eV. This shift suggests that nitrogen atoms participate in hydrogen bonding, which reduces their electron density [47]. This further confirms the presence of strong non-covalent bonding between CNS and PAM, forming a hydrogen-bonded network.

As shown in Figure 4c and Figure S4, FTIR and Raman spectroscopy were used to further analyze the changes in the hydrogen-bonding network in the sand-fixing agent. The relative content of intermolecular hydrogen bonds in cellulose increased from 58.07% to 63.92%, while intramolecular hydrogen bonds decreased from 38.71% to 27.72% (Figure S4a). This confirms the crosslinking between the -NH_2_ in PAM and -COO^−^ on the CNS surface. The hydrogen bonds between -COO^−^ and -CONH_2_ are stronger than those formed with water molecules, leading to a rearrangement of the hydrogen bonds and resulting in a denser and more directional network. The proportion of ordered hydrogen bonds increased from 22.81% to 29.01% (Figure S4b), which further supports the enhanced stability of the network. After composite formation, the proportion of strong hydrogen bonds in CNS increased from 18.55% to 26.08%, and the moderate hydrogen bond ratio from 41.53% to 44.77%, while weak hydrogen bond decreased from 39.91% to 29.91%. The increase in strong hydrogen bonds indicates dense intermolecular interactions between the carboxylated cellulose and the matrix [48]. At the same time, the shift in characteristic peaks to lower wavenumbers suggests that molecular chains rearrange due to the formation of stronger hydrogen bonds. Compared with pure PAM, the proportion of strong hydrogen bonds decreased from 39.03% to 26.06%, while medium-strength hydrogen bonds increased from 34.44% to 44.77% after composite formation. This indicates strong intermolecular interactions between CNS and PAM, which break the original strong hydrogen bonds and rebuild a more uniform and stable supramolecular network dominated by medium-strength hydrogen bonds.

As shown in Figure 4d, after centrifugation at 8000 r/min for 20 min, CNS forms a gel-like sediment, while the PN solution remains stable with no visible sediment. Rheological tests (Figure 4e) show that all samples exhibit shear-thinning behavior, which is suitable for large-scale spraying [49]. The viscosity of the composite solution increased significantly to 5287.2 mPa·s, further proving the stable structure of the composite solution.

Figure 4f illustrates the supramolecular assembly process. The surface molecular chains of CNS contain a large amount of -OH and -COOH/-COO^−^, while the PAM molecular chains are rich in -CONH_2_, which are ideal sites for hydrogen bonding. After recombination, a denser intermolecular hydrogen-bonding network is formed between CNS and PAM. The proportion of intramolecular hydrogen bonds is relatively reduced.

### 3.4. Sand Stabilization Structure and Performance

**Figure 5:**
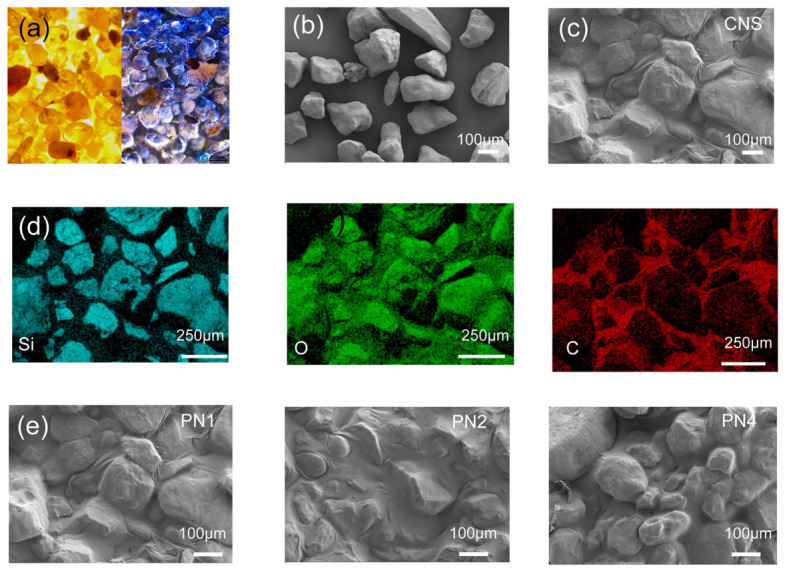
Sand fixation morphology. (**a**) Optical images of gravel surface before and after sand-fixing agent spraying; (**b**,**c**) SEM images of the gravel surface before and after sand-fixing agent spraying; (**d**) EDS image of gravel surface after sand-fixing agent spraying; (**e**) SEM images of the gravel surface after spraying different composite sand-fixing agents.

The surface morphology of the sand before and after spraying the sand-fixing agent is shown in Figure 5a. The untreated sand is loosely packed. After spraying, a continuous and transparent consolidation film forms on the surface. This film wraps and fixes the dispersed particles at the macroscopic level and clearly improves the integrity of the surface layer. As shown in Figure 5b, there are many gaps between untreated sand particles, making them easy to move under external forces such as wind and water. After treatment, a continuous consolidation layer forms on the particle surface. This layer not only fills the gaps between particles but also builds stable connections among them (Figure 5c). EDS analysis shows that this layer is uniformly distributed on the sand surface. It forms bridge-like structures at particle contact areas and acts as an interfacial binder (Figure 5d). As shown in Figure 5e, after combining with polyacrylamide (PAM), the surface layer remains continuous and intact. This indicates that the introduction of PAM does not damage the layered structure formed by the two-dimensional nanosheets. Instead, it further improves the integrity and resistance to damage of the layer by strengthening the hydrogen-bonding network and adding molecular chain entanglement.

**Figure 6:**
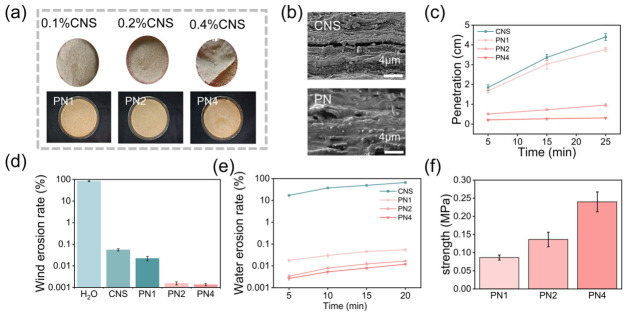
Sand fixation performance. (**a**) Different film-forming structures of sand-fixing agents; (**b**) structure network of sand-fixing agent before and after composite; (**c**) the permeability of different sand-fixing agents; (**d**) different sand-fixing agents have the ability to resist wind erosion; (**e**) anti-water erosion ability of different sand-fixing agents: (**f**) compressive strength of sand cores with different sand-fixing agents.

As shown in Figure 6a, the consolidation layer remains intact as the concentration of the sand-fixing solution increases. However, when the content of cellulose nanosheets reaches 0.4%, a clear interfacial separation appears between the consolidation layer and the underlying sand. This result indicates that a single two-dimensional nanosheet system can form a dense film at high concentration, but the strong interactions between nanosheets increase internal cohesion and weaken their ability to adapt to the rough particle surface. After adding PAM, its linear chains form flexible connections both between nanosheets and at the interface with sand particles. On one hand, chain entanglement and hydrogen bonding regulate the interactions between nanosheets and prevent excessive stacking. On the other hand, the bridging effect of PAM spans the gaps between nanosheets and sand particles, which effectively suppresses the overall delamination of the consolidation layer.

As shown in Figure 6b, in different permeation networks, the single-sheet system forms gaps between particles, while after composite with PAM, the interlayer pores are effectively filled, leading to closer contact with the particles and improved overall permeability and structural stability [50]. Figure 6c presents the infiltration behavior of different sand fixatives within 25 min. The penetration depth of the 0.1% CNS solution reached 4.4 cm. After compounding with 0.2% PAM, the penetration performance decreased slightly to 3.77 cm. When the CNS content in the composite system was 0.4%, the penetration depth drops to about 0.33 cm. This trend indicates that as the concentration of the sand-fixing solution increases, the hydrogen-bonding network becomes denser, making it more difficult for the solution to enter the pores between sand particles [51]. This result shows that the formation of the hydrogen-bonding network limits the flow and penetration ability of the solution, which affects its penetration depth and distribution.

To evaluate sand-stabilization performance, wind erosion, water erosion, and compressive strength were systematically tested [52]. As shown in Figure 6d, the sand treated with pure water shows rapid mass loss under wind erosion, with a loss rate as high as 83.5%. After applying the sand-fixing agent, CNS can effectively reduce wind-induced sand loss at a low concentration (0.1%), with a loss of less than 0.1%. With the incorporation of 0.2 wt% PAM, wind erosion resistance was further improved (reduction > 99.9%), indicating that the composite system shows a synergistic enhancement in interfacial stability and structural integrity. As the content of nanosheets increases, the ability of the system to bind and wrap sand particles continues to improve, leading to better resistance to wind erosion.

In addition, a device shown in Figure S5a was used to measure water erosion resistance. As shown in Figure 6e and Figure S5b, the sand consolidated with a single nanosheet dispersion shows limited resistance to water erosion, with a mass loss of up to 21% within 5 min. This indicates that the structure is easily damaged under liquid disturbance. After adding PAM, the water erosion resistance improves significantly. Under a condition of 10 L/m^2^, the sand shows almost no mass loss within 15 min, and the shape remains intact, indicating effective stabilization. This behavior occurs because, although the nanosheet can form a continuous layer on the sand surface, the relatively large sheet size limits its penetration into the particle packing. As a result, the consolidation layer mainly forms on the surface, with weak structural connection to the underlying sand (Figure 6a,b) [53]. During the water infiltration, the liquid preferentially enters the weak interfacial regions, causing local structural relaxation and collapse, which eventually leads to the detachment and failure of the whole consolidation layer.

After adding PAM, the water erosion resistance performance is significantly improved. On one hand, the higher viscosity of the PAM solution enhances its penetration and retention within the gaps between sand particles, which improves interfacial contact between the consolidation layer and the underlying sand. On the other hand, the linear molecular chains of PAM form bridging connections between the two-dimensional nanosheets and sand particles, creating a multiscale network that compensates for the limited vertical structural connection in the single-nanosheet system [54].

The slaking resistance was evaluated by immersing the samples in water (Figure S6). In the system without PAM, the water in the beaker is initially clear but gradually turns light brown as some sand particles detach and disperse into the water. With increasing immersion time, the volume of the sand cone decreases, and the overall structure is eventually destroyed. In contrast, after adding PAM, the sand pile remains nearly unchanged even after five days of immersion, showing that the consolidation layer maintains good mechanical stability in a liquid environment. This further confirms that PAM forms a dense network through its penetration effect and creates multiscale bridging between cellulose nanosheets and sand particles, thereby enhancing mechanical strength in the vertical direction.

The compressive strength of the consolidated sand columns was evaluated through compression tests (Figure 6f). The CNS solution primarily formed a surface crust on the sand, while the inner sand core is poorly formed and shows almost no compressive strength. In contrast, adding PAM improved the molding ability of the sand due to the enhancement of the overall hydrogen bond network in the solution.

The linear molecular chains of PAM not only interacted with cellulose nanosheets via hydrogen bonding, but also formed flexible connections between the CNS and the sand surfaces. This bridging effect effectively improved the bonding strength between sheets and reduced excessive material accumulation, making the overall structure of the consolidated sand column more compact and stable [55]. As the sheet content increased, the compressive strength gradually rose from 0.08 MPa to 0.23 MPa.

Furthermore, a regression analysis was performed to examine the relationship between concentration and strength. The results show a strong positive linear correlation. The data points are closely distributed around the fitted line, and the coefficient of determination (R^2^) reaches 0.99, indicating that the linear model fits the data very well (Figure S7).

In addition, the sand-fixing agent shows good wettability. As shown in Figure S8, the water contact angle of different films was measured. CNS has the lowest contact angle of 67.8°. After adding PAM, the contact angle increases. However, with increasing CNS content, the number of carboxyl groups rises, and the contact angle decreases again. All films show hydrophilic behavior [56].

### 3.5. Analysis of Sand Fixation Mechanism

**Figure 7:**
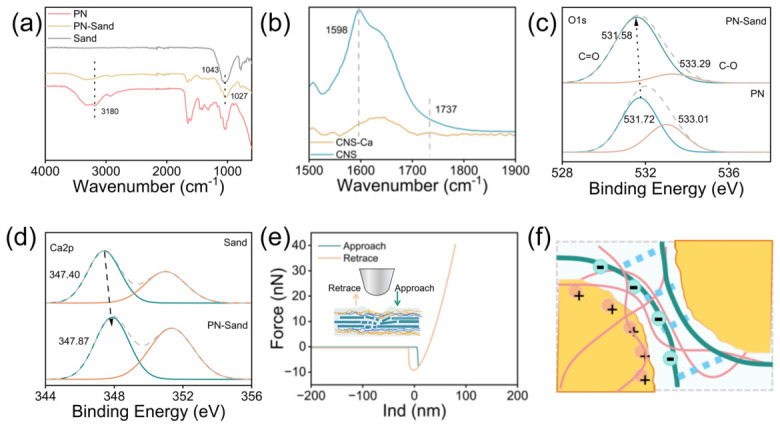
The sand-fixing agent interacts with the gravel. (**a**) FTIR spectra of PN before and after spraying sand fixing solution; (**b**) infrared spectra of carboxyl coordination with metal ions; (**c**,**d**) coordination between PN and gravel; (**e**) microscopic adhesion of CNS; (**f**) schematic diagram of the interaction between sand-fixing agents and gravel surfaces.

Chemical interactions govern both interfacial anchoring and colloidal stability. As shown in Figure 7a, the pure sand exhibited no obvious absorption peaks in the 3000–3500 cm^−1^ region. In contrast, distinct characteristic peaks appeared in this region after compounding. These peaks are attributed to the -OH and -NH stretching vibrations of the sand-fixing agents (PAM/cellulose nanosheets). This confirms the successful loading and coating of the agents on the gravel surface.

After mixing with the gravel, the -NH peak at 3180 cm^−1^ showed significant broadening. At the same time, the asymmetric stretching peak of sand (Si–O–Si) at around 1043 cm^−1^ shifts to a lower wavenumber, moving to 1027 cm^−1^ [57]. These spectral changes clearly indicate that intermolecular hydrogen bonds form between the polar groups (-CONH_2_) on the sand-fixing agent and the silanol groups (Si–OH) or oxygen atoms on the sand surface. This interaction enables effective bonding and coating of the sand particles by the sand-fixing agent.

In addition to hydrogen bonding, the surface of CNS, rich in carboxyl groups, forms coordination interactions with metal cations on the sand surface.

Figure S9a shows the XPS spectrum of the sand sample, where characteristic peaks of metal elements such as Ca and Al are observed. This indicates that the sand surface contains abundant coordination sites. Figure 7b shows the coordination interaction between CNS and inorganic metal ions. The peak at 1596 cm^−1^, assigned to carboxylate groups, decreases significantly, and a new peak appears at 1737 cm^−1^. This confirms that carboxyl groups form coordination and chelation interactions with metal ions on the mineral surface [58]. To further study the interaction mechanism, XPS was used to analyze the coordination changes. As shown in Figure 7c, the O 1s spectrum was deconvoluted. In the pure sand-fixing agent, the peak assigned to carboxyl/carbonyl (C=O) appears at 531.72 eV. In the sand-fixing agent–sand mixture, this peak shifts to a lower binding energy of 531.58 eV. This decrease indicates an increase in electron density around the oxygen atoms. This is because the oxygen atoms in carboxyl and amide groups act as electron donors and form coordination bonds with metal cations (Ca^2+^/Al^3+^) on the sand surface [59]. In addition, in the C 1s spectrum, the C4 peak represents the carboxyl groups in cellulose nanosheets. After mixing with sand, its binding energy decreases from 289.01 eV to 288.23 eV (Figure S9b). In the Ca spectrum, the binding energy increases from 347.40 eV in pure sand to 347.87 eV after mixing (Figure 7d). These results demonstrate that the composite sand-fixing agent forms stable coordination complexes with metal ions on the sand surface through intermolecular interactions. This enhances the bonding between the agent and sand particles and improves its environmental stability and sand-fixing performance.

In addition, based on the surface composition of CNS, atomic force microscopy (AFM) was used to evaluate the microscale adhesion properties of the material (Figure 7e). Under a load of 50 nN, three points were measured at each location, and the surface adhesion force reaches up to 15 nN. This strong adhesion mainly comes from its unique multi-component biomass structure. In terms of composition, the material retains the rigid cellulose framework while still containing a certain amount of amorphous polysaccharides such as hemicellulose, which provide natural adhesion. After oxidation of the cellulose framework, active groups such as carboxyl groups are introduced on the surface, which significantly enhance interfacial interactions. At the same time, functional groups in residual lignin, such as phenolic hydroxyl and methoxy groups, can coordinate with metal ions in the environment. This forms a dynamic coordination network at the interface and further strengthens the adhesion of the nanosheets [60,61].

Figure 7f illustrates the interaction between the sand-fixing agent and sand. On one hand, the physical adhesion of the cellulose nanoflakes and the hydrogen bond network effectively aggregate the sand. On the other hand, the electrostatic coordination between the cellulose nanoflakes and the sand surface allows for good dynamic adaptation to different surface morphologies, thereby enhancing structural stability.

### 3.6. Sustainable Performance

**Figure 8:**
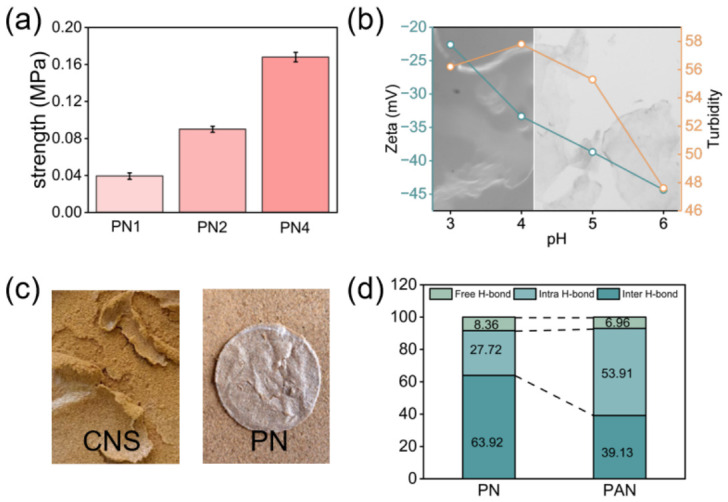
Aging performance of sand-fixing agent. (**a**) Compressive performance after UV aging; (**b**) aggregation of CNS solution under acid aging; (**c**) film integrity of different sand-fixing agents under acid aging; (**d**) the hydrogen-bonding network changes in PN and acid-aged sand-fixing agent (PAN).

To evaluate sustainability, UV and chemical-aging tests were conducted. As shown in Figure 8a, UV aging led to a reduction in compressive strength (PN1: 0.04 MPa; PN2: 0.09 MPa; PN4: 0.16 MPa). This is attributed to UV-induced chain scission in cellulose and PAM, which lowers the degree of polymerization and disrupts the hydrogen bond network. Additionally, prolonged UV exposure may promote the formation of high-energy free radicals, further exacerbating chain breakage and the degradation of the cross-linked structure [62,63].

Citric acid was used to acidify the dispersion for the aging treatment. Due to the carboxylate groups on the CNS surface, a disordered gel network formed under acidic conditions, driven by strong intermolecular electrostatic interactions. The sheet suspension gradually aggregated or precipitated, and obvious dehydration occurred in the solution (Figure 8b) [64,65]. Zeta potential was measured via potentiometric titration. As shown in Figure 8b, the zeta potential of the sheet dispersion decreased from −42 mV to −22 mV, indicating that the dispersion became less stable as the pH decreased. Turbidity measurements also showed that with the addition of acid, the volume of the sheet polymers in the dispersion increased. This suggests a weakening of the electrostatic repulsion between the sheets, making the solution increasingly unstable. Consequently, aggregation driven by interlayer forces and stacking tendency is unfavorable for sand fixation.

However, after the addition of PAM, the system (PAN) remained stable under acidic conditions. The linear structure of PAM molecules provided strong steric hindrance, which effectively prevented particle aggregation and phase separation. The composite system (PAN) maintained a uniform state without any obvious separation. This indicates that the flexible PAM chains not only stabilize the hydrogen bond network under acidic conditions but also provide additional mechanical stability to the dispersion. This prevents structural damage to the sand-fixing liquid in acidic environments, thereby ensuring the integrity of the consolidated layer (Figure 8c).

Infrared spectroscopy was used to study the hydrogen bond structure after acid aging. As shown in Figure S10, the proportion of intermolecular hydrogen bonds in the PAN system decreased sharply. Specifically, as shown in Figure 8d, it drops from 63.92% to 39.13%, while the proportion of intramolecular hydrogen bonds increases from 27.72% to 53.91%.

Acid aging causes the -COO^−^ groups on carboxylated cellulose to be protonated to -COOH, which weakens their ability to act as hydrogen bond acceptors [48]. At the same time, the acidic environment disrupts the compatible network between PAM and cellulose nanosheets, leading to the breakdown of intermolecular hydrogen bonds. However, compared with carboxyl groups, amide groups are relatively stable in weakly acidic to neutral conditions. Therefore, with the bridging effect of PAM, the overall stability of the composite solution remains higher than that of the single CNS system.

### 3.7. Environmental Compatibility and Biodegradation Behavior

**Figure 9:**
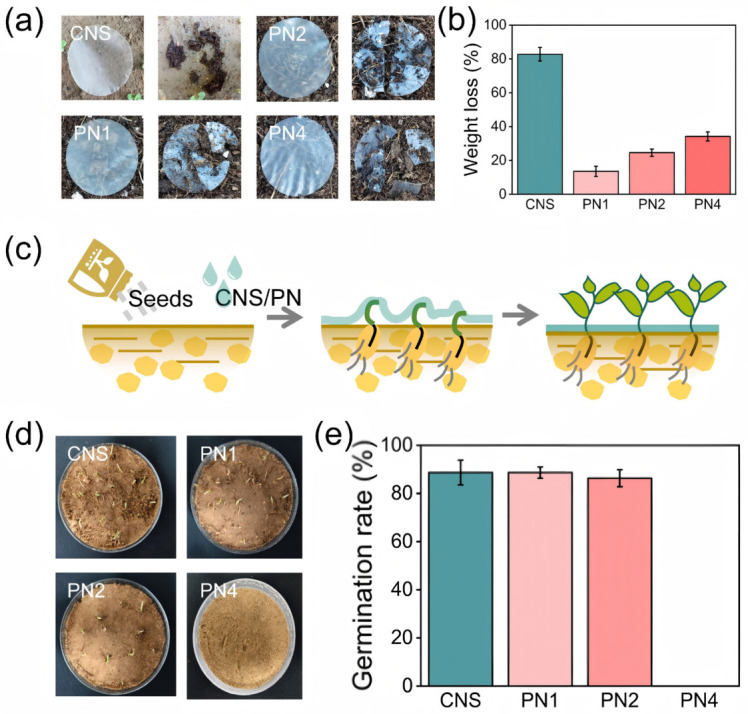
Ecological friendliness of sand-fixing agents. (**a**) Degradation images of different sand-fixing agent films within 45 days; (**b**) degradation quality loss of different sand-fixing agent films within 45 days; (**c**) indications of seed growth obstacles; (**d**) seed germination status; (**e**) seed germination rate.

To further evaluate the eco-friendliness of the sand-fixing agent, degradation tests were carried out on different films. As shown in Figure 9a, the pure CNS film loses its structure completely after 45 days of soil degradation due to the action of water, enzymes, and other factors. This indicates that the cellulose chains are largely broken. The mass loss reaches up to 82.7%, and most components are degraded into carbon dioxide and water, confirming its biodegradability [66,67]. After adding PAM, the degradation rate decreases. The mass loss of PN1 is only 13.56%, PN2 is 24.63%, and PN4 is 34.2%. As the CNS content increases, the mass loss gradually increases (Figure 9d). After combining with PAM, only local cracks and pores appear on the film surface. This suggests that partial degradation of cellulose leads to the breakage between cellulose and PAM chains, while the overall layered framework of the film remains intact, effectively extending its service time for sand fixation.

Increasing the concentration of the sand-fixing agent can improve the stabilization of gravel, but it may also increase the hardness of the consolidation layer, which can negatively affect plant growth. This may reduce the ecological benefits in practical use. As shown in Figure 9c, different concentrations of the sand-fixing agent were applied to study plant growth. At an application rate of 3 L/m^2^, both 0.1% and 0.2% CNS show no effect on seed germination. After combining with PAM, PN1 and PN2 also show no negative effects, with germination rates above 80%. However, under the PN4 condition, the high-strength consolidation layer strongly inhibits seed growth, and no germination is observed within the same period (Figure 9e).

To further evaluate the potential application safety of the composite sand-fixing agent, its cytotoxicity was tested. In vitro experiments were conducted using human renal cortical proximal tubule epithelial cells (HK-2). Figure S11 displays the cell viability at concentrations of 0 μg/mL and 100 μg/mL. Cell survival was imaged using laser confocal microscopy. MTT assay results showed that after 24 h of incubation, no significant cell death occurred at various concentrations [68]. These results suggest that the composite sand-fixing agent is non-toxic and safe for the human body, exhibiting excellent biocompatibility.

## 4. Discussion

In conclusion, this study presents a bio-inspired, cellulose-based sand fixation material that leverages an interface coating strategy derived from plant cell wall structure. By combining CNS with PAM, the hydrogen-bonding network is effectively regulated, leading to a composite with strong resistance to wind and water erosion (erosion reduction > 99%), a compressive strength of up to 0.23 MPa, and good structural stability during long-term water immersion.

The improved performance comes from the synergistic effect of nanoscale interfacial interactions and a dynamic supramolecular hydrogen-bonding network. CNS acts as the main fixing component, enabling effective wrapping and interlocking of sand particles through polysaccharide adhesion, coordination with mineral ions, and interfacial hydrogen bonding. The addition of PAM further reorganizes the hydrogen-bonding network among nanosheets, polymer chains, and confined water, which enhances structural adaptability and maintains stability under acidic conditions. This dynamic non-covalent network provides good mechanical strength while improving environmental tolerance. Together with good wettability and natural biodegradability, the composite system achieves a balance between performance, environmental compatibility, and sustainability. Overall, this work offers a promising and sustainable approach for sand fixation and desertification control.

## Data Availability

The original contributions presented in this study are included in the article. Further inquiries can be directed to the corresponding author.

## Supplementary Materials

The following supporting information can be downloaded at: https://www.mdpi.com/article/10.3390/polym18101188/s1.
