# Supplementary material for: Supramolecular Assembly of Plant Cell Wall-Derived Cellulose Nanosheets with Polyacrylamide for Sustainable Sand Stabilization
Source: Polymers (Basel). 2026 May 13;18(10):1188. doi: 10.3390/polym18101188 (PMC13211247; doi:10.3390/polym18101188)
Supplement: Supplementary file 1 [file polymers-18-01188-s001.zip › polymers-4292899-supplementary.pdf]

Supporting Information

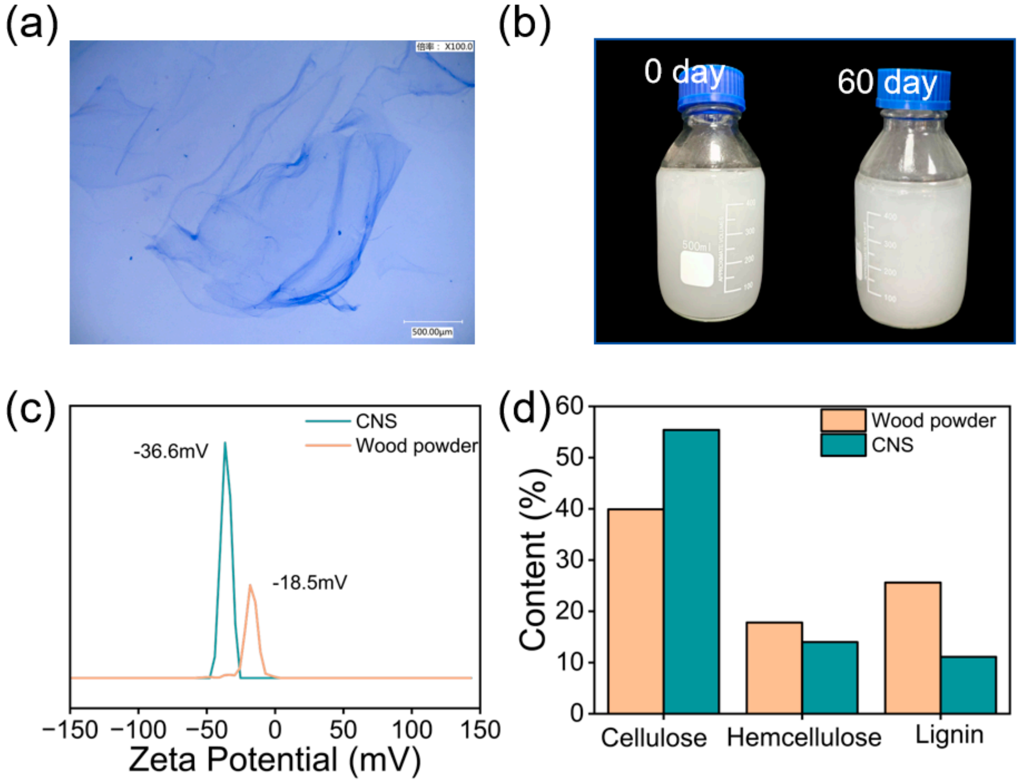

Figure S1. (a) Light microscope image of CNS dispersion; (b) Stability of CNS dispersion; (c) Zeta potential of CNS dispersion and zeta potential of the original powder; (d) Chemical composition of CNS.

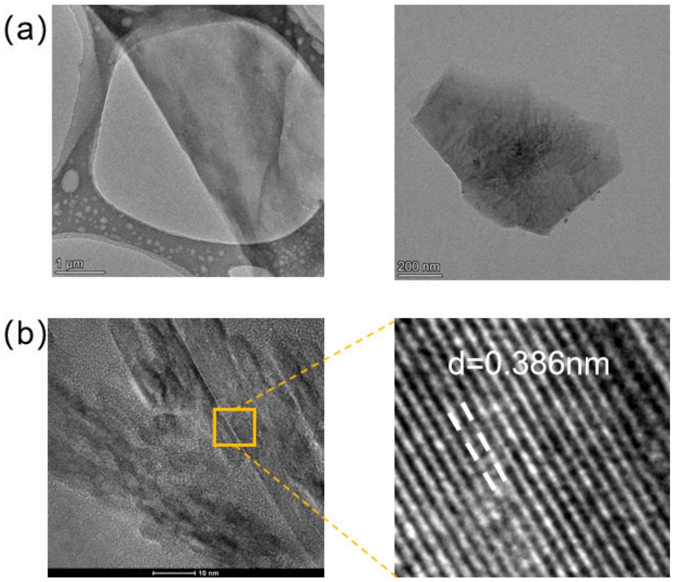

Figure S2. (a) TEM images of CNS; (b) Crystal structure of CNS

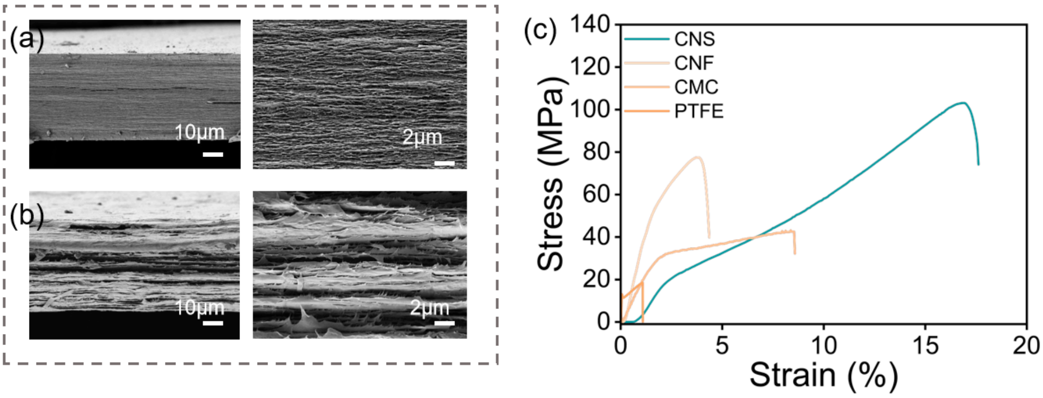

Figure S3. (a) Cross section SEM of CNF thin film; (b) CNS thin film cross-section SEM; (c) Comparison of CNS thin film mechanics

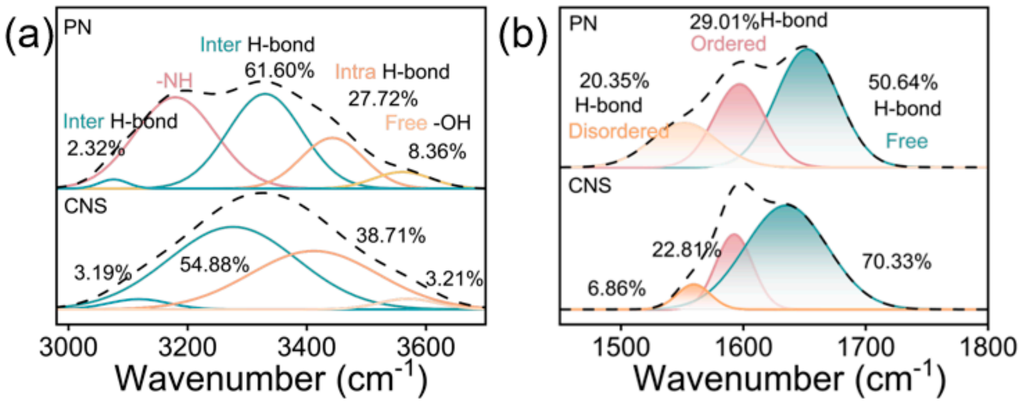

Figure S4. (a) CNS and PN exhibit infrared peaks at 3000-3800cm^{-1}; (b) CNS and PN exhibit infrared peaks at 1500-1800cm^{-1}.

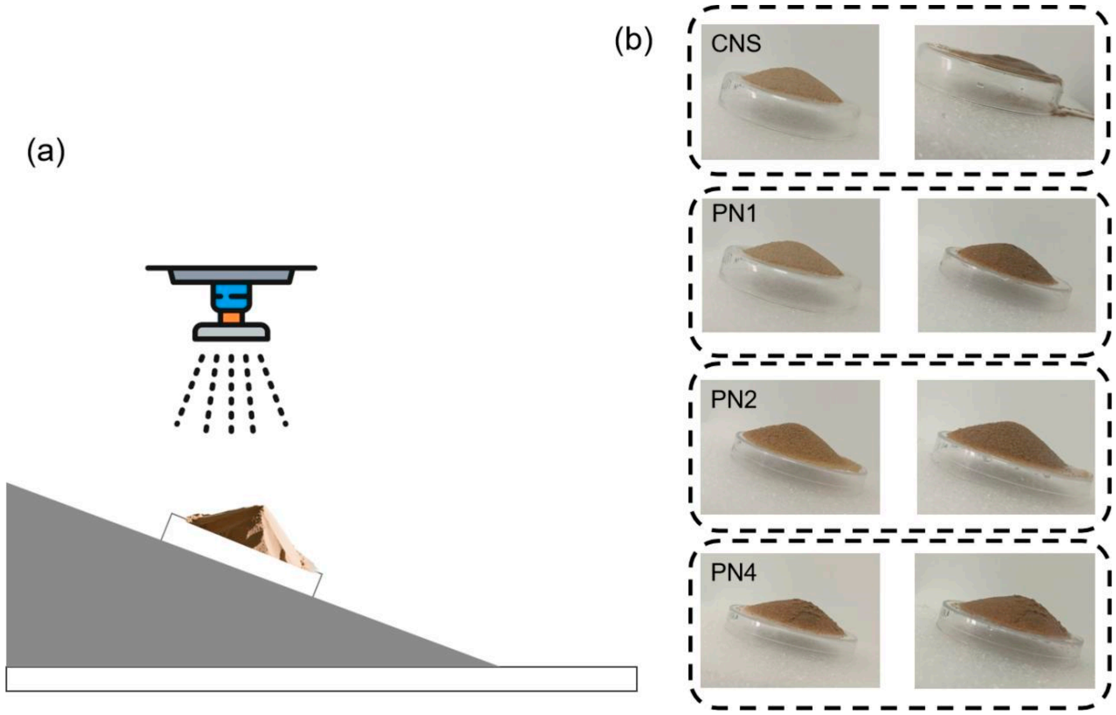

Figure S5. (a) Intention of water erosion laboratory; (b) Images before and after 15 minutes of water erosion with different sand fixing agents.

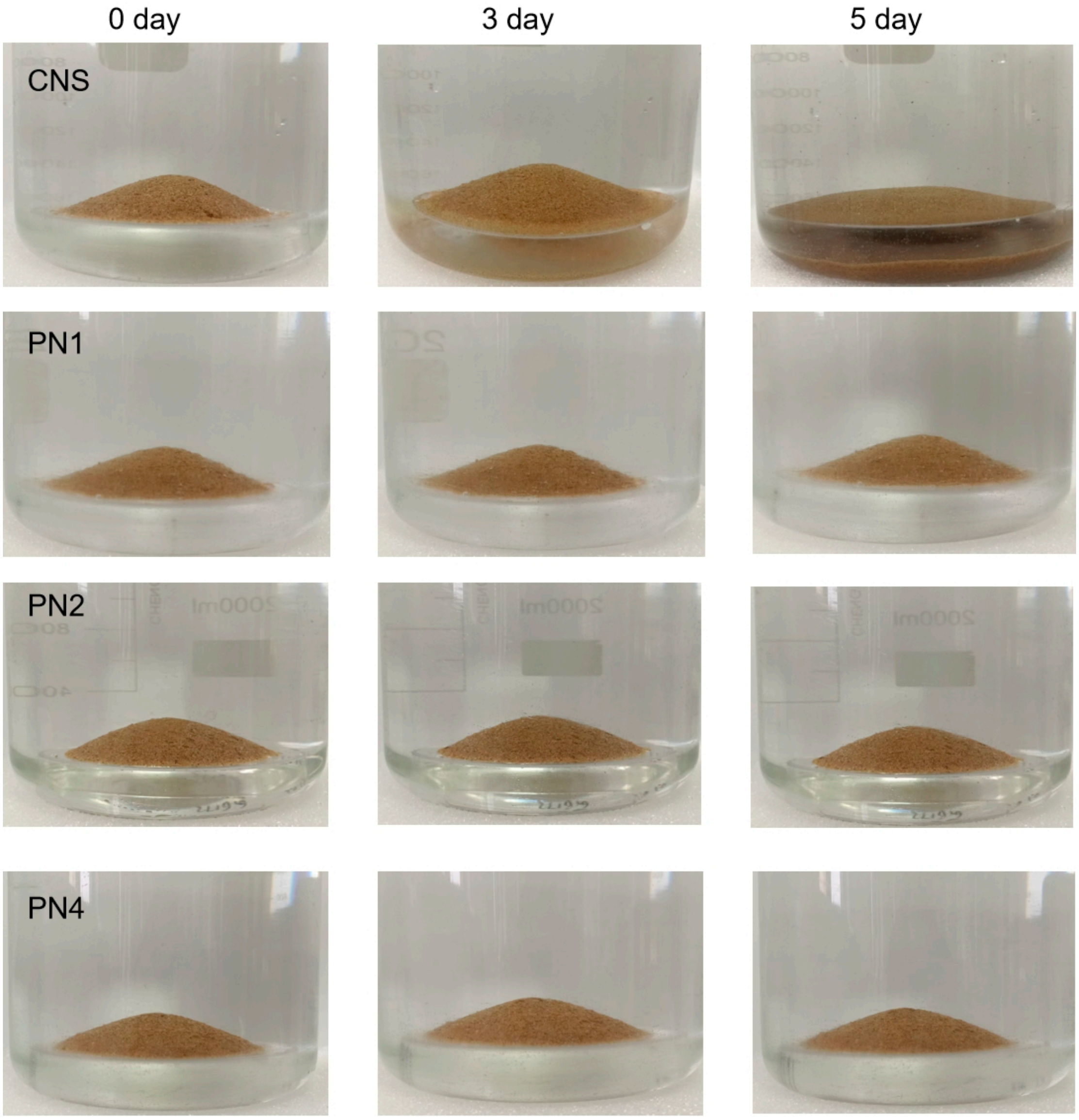

Figure S6. Resistance of different sand fixing agents to water immersion and disintegration.

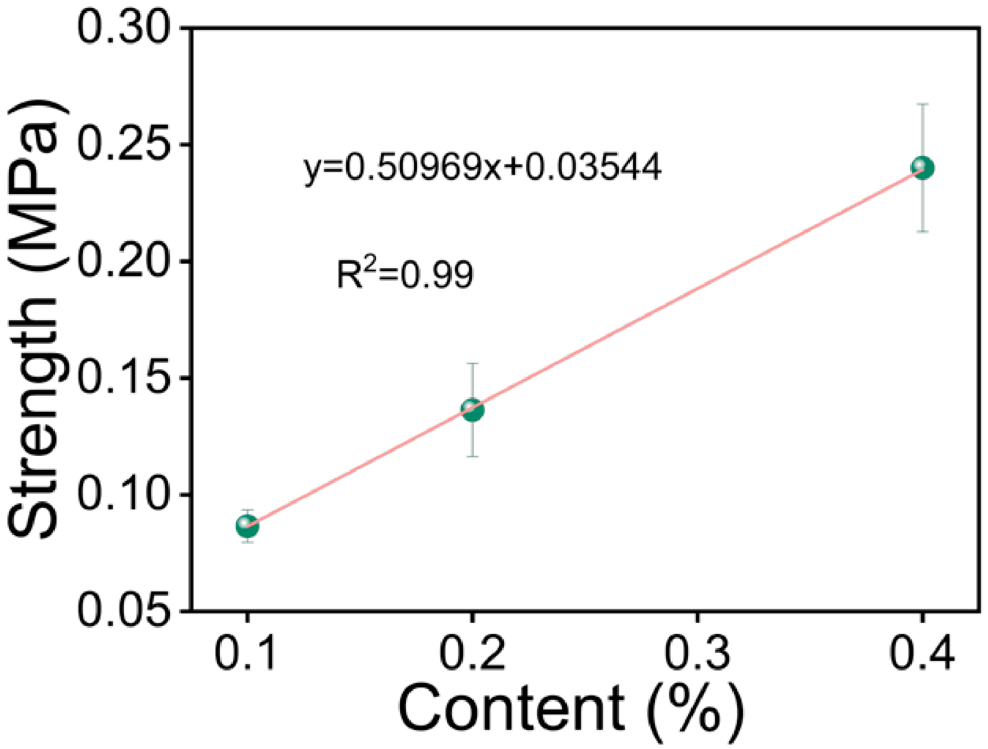

Figure S7.Linear fitting of CNS content and strength in composite sand fixing agents

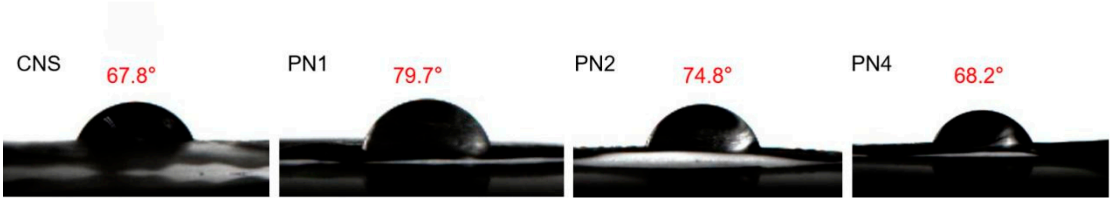

Figure S8. Water contact angle of different sand fixing agents.

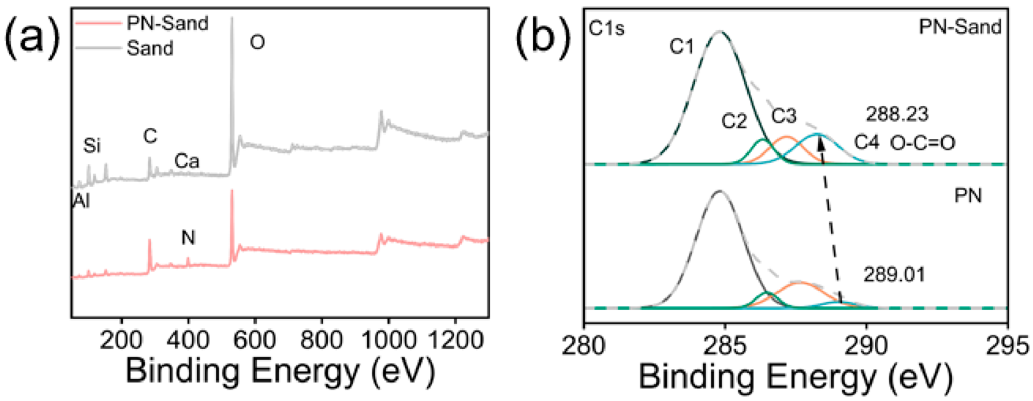

Figure S9. (a) XPS spectra of sand and gravel after spraying with sand fixing agent; (b) Peak distribution of C-spectus before and after spraying composite sand fixing agent on gravel

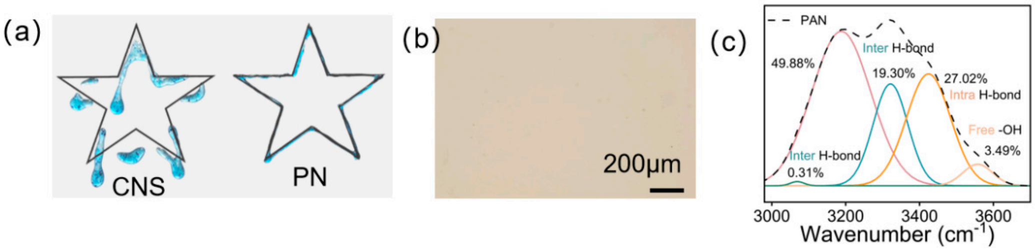

Figure S10. (a) Stability of CNS and PN solutions under acid aging conditions; (b) Optical microscopy image of PN solution under acid aging conditions; (c) Hydrogen bond network peak of PAN sand fixing agent.

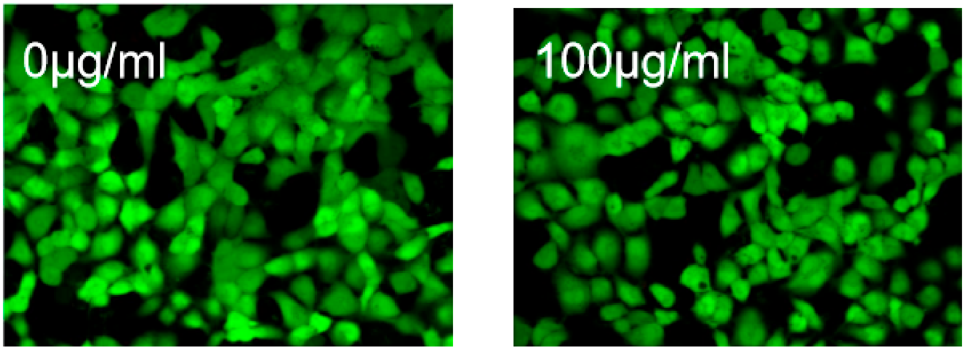

Figure S11. Cell Live/Dead staining laser confocal images of HK-2 cells co cultured with samples at different concentrations for 24 hours.
